# Essential Role of the EF-hand Domain in Targeting Sperm Phospholipase Cζ to Membrane Phosphatidylinositol 4,5-Bisphosphate (PIP_2_)*

**DOI:** 10.1074/jbc.M115.658443

**Published:** 2015-10-01

**Authors:** Michail Nomikos, Jessica R. Sanders, Dimitris Parthimos, Luke Buntwal, Brian L. Calver, Panagiotis Stamatiadis, Adrian Smith, Matthew Clue, Zili Sideratou, Karl Swann, F. Anthony Lai

**Affiliations:** From the ‡ Institute of Molecular and Experimental Medicine, School of Medicine, Cardiff University, Cardiff CF14 4XN, United Kingdom and; the § National Center for Scientific Research “Demokritos,” 15310 Aghia Paraskevi, Greece

**Keywords:** calcium intracellular release, fertilization, phosphatidylinositol signaling, phospholipase C, sperm, EF-hand domain, PIP2, inositol 1,4,5-trisphosphate

## Abstract

Sperm-specific phospholipase C-ζ (PLCζ) is widely considered to be the physiological stimulus that triggers intracellular Ca^2+^ oscillations and egg activation during mammalian fertilization. Although PLCζ is structurally similar to PLCδ1, it lacks a pleckstrin homology domain, and it remains unclear how PLCζ targets its phosphatidylinositol 4,5-bisphosphate (PIP_2_) membrane substrate. Recently, the PLCδ1 EF-hand domain was shown to bind to anionic phospholipids through a number of cationic residues, suggesting a potential mechanism for how PLCs might interact with their target membranes. Those critical cationic EF-hand residues in PLCδ1 are notably conserved in PLCζ. We investigated the potential role of these conserved cationic residues in PLCζ by generating a series of mutants that sequentially neutralized three positively charged residues (Lys-49, Lys-53, and Arg-57) within the mouse PLCζ EF-hand domain. Microinjection of the PLCζ EF-hand mutants into mouse eggs enabled their Ca^2+^ oscillation inducing activities to be compared with wild-type PLCζ. Furthermore, the mutant proteins were purified, and the *in vitro* PIP_2_ hydrolysis and binding properties were monitored. Our analysis suggests that PLCζ binds significantly to PIP_2_, but not to phosphatidic acid or phosphatidylserine, and that sequential reduction of the net positive charge within the first EF-hand domain of PLCζ significantly alters *in vivo* Ca^2+^ oscillation inducing activity and *in vitro* interaction with PIP_2_ without affecting its Ca^2+^ sensitivity. Our findings are consistent with theoretical predictions provided by a mathematical model that links oocyte Ca^2+^ frequency and the binding ability of different PLCζ mutants to PIP_2_. Moreover, a PLCζ mutant with mutations in the cationic residues within the first EF-hand domain and the XY linker region dramatically reduces the binding of PLCζ to PIP_2_, leading to complete abolishment of its Ca^2+^ oscillation inducing activity.

## Teaser

**Background:** The mechanism underlying sperm PLCζ interaction with its target membrane is unresolved.

**Results:** EF-hand mutations introduced into PLCζ reduce *in vivo* Ca^2+^ oscillation inducing activity and *in vitro* interaction with PIP_2_.

**Conclusion:** EF-hand domain is essential for targeting PLCζ to PIP_2_-containing membranes.

**Significance:** We propose a novel mechanism by which sperm PLCζ is anchored to its physiological membrane substrate.

## Introduction

During fertilization, the spermatozoon initiates activation of egg development by triggering an acute rise in cytosolic free Ca^2+^ concentration (1). In mammals, this manifests as a series of distinctive cytosolic Ca^2+^ oscillations, beginning soon after sperm-egg fusion and persisting for several hours (2). The weight of evidence now suggests that Ca^2+^ oscillations appear to be caused by a sperm-specific protein, phospholipase C-ζ (PLCζ),^3^ which is introduced into the egg upon sperm-egg fusion and leads to cycles of inositol 1,4,5-trisphosphate (IP_3_) production following PIP_2_ hydrolysis, thus activating IP_3_ receptor-mediated Ca^2+^ release from intracellular stores in the egg (3–12). The closest PLC homologue of sperm PLCζ is PLCδ1 (47% similarity, 33% identity), which is only able to cause Ca^2+^ oscillations in mouse eggs at non-physiological concentrations, because it has a >50-fold lower potency (2, 3, 12). The superior fertilization potency of the sperm PLCζ over somatic PLCs has not yet been fully explained.

PLCζ is the smallest PLC with the simplest domain organization among all the mammalian isoforms. PLCζ consists of four tandem EF-hand domains, the characteristic X and Y catalytic domains in the center of the molecule, and a C-terminal C2 domain. All these domains are common to the other PLC isoforms (β, γ, δ, ϵ, and η), but they appear to individually have an essential role in the unique mode of regulation of this distinctive PLC isozyme (2). A notable structural difference between PLCζ and the other somatic PLC isoforms is that PLCζ lacks a pleckstrin homology (PH) domain at the N terminus (2, 3, 13). The membrane binding of somatic PLCs appears to be mediated by the PH domain, a well defined structural module of ∼120-amino acid residues identified in numerous proteins (14). The PH domain of PLCδ1 is essential for interaction with its phospholipid substrate PIP_2_ in the plasma membrane (15). The absence of a PH domain from PLCζ sequence raises questions about how PLCζ can bind to membranes.

We have previously proposed that the PLCζ XY-linker, a segment between the X and Y catalytic domains that is notably different from the corresponding XY-linker region of somatic PLCs, is involved in the targeting of PLCζ to its membrane-bound substrate PIP_2_ (16, 17). The XY-linker region of PLCζ is extended in length and consists of more basic residues relative to its PLCδ1 counterpart. The affinity of the XY-linker for PIP_2_ appears to involve a polybasic charged region that is found in a number of other membrane-associated proteins (16, 18). These positively charged amino acids in the XY-linker appear to assist the anchoring of PLCζ to membranes by enhancing the local PIP_2_ concentration adjacent to the XY catalytic domain via electrostatic interactions with the negatively charged PIP_2_ (16, 17). However, the XY-linker might not be the only domain that mediates the binding of PLCζ to PIP_2_-containing membranes. We have demonstrated that the absence of the XY-linker from PLCζ significantly diminishes, but does not completely abolish, the *in vivo* Ca^2+^ oscillation inducing activity (19). This suggests that other domain(s) may also be involved in anchoring PLCζ to its target membrane.

A recent study reported that the N-terminal lobe of the EF-hand domain of PLCδ1 binds anionic phospholipids, and this binding is due to interactions with cationic and hydrophobic residues in the first EF-hand sequence of PLCδ1 (20). The authors propose a general mechanism that may apply to other PLC isoforms by suggesting that EF-hand domain interactions with anionic phospholipids in the target membrane provides a tether that facilitates proper substrate access and binding in the active site (20). Importantly, the cationic residues in the first EF-hand domain of PLCδ1 that contribute to anionic lipid vesicle binding are all conserved in PLCζ.

The aim of this study is to investigate the potential importance of a conserved cluster of cationic residues at the N-terminal lobe of the EF-hand domain of PLCζ in association with anionic lipids and its substrate PIP_2_. A series of full-length mouse PLCζ mutants were prepared that sequentially neutralized two positively charged lysine and one arginine residues within the first EF-hand domain. The Ca^2+^ oscillation-inducing properties of these mutants were experimentally tested relative to wild-type PLCζ by microinjection of cRNA into unfertilized mouse eggs. The various PLCζ mutants' enzymatic properties were analyzed using an *in vitro* PIP_2_ hydrolysis assay. A protein-lipid overlay and a liposome binding/enzyme assay were employed to assess the binding properties of wild-type PLCζ to phosphatidylserine (PS), phosphatidic acid (PA), and PIP_2_. Furthermore, the binding properties of mutant EF-hand PLCζ proteins to PIP_2_ were examined. Our results suggest that PLCζ possesses significant affinity only for PIP_2_ but not for PA or PS. We also find that sequential reduction of the net positive charge within the first EF-hand domain significantly reduces both *in vivo* Ca^2+^ oscillation inducing activity and the *in vitro* interaction of PLCζ with PIP_2_. Moreover, we show that a PLCζ mutant where three cationic residues within the first EF-hand domain and three cationic residues within the XY-linker region of PLCζ were substituted by alanine is unable to trigger Ca^2+^ oscillations in mouse eggs. *In vitro* biochemical characterization suggests that this PLCζ mutant displays dramatically reduced binding to PIP_2_-containing liposomes compared with the wild-type PLCζ. Thus, we propose a novel mechanism for the sperm PLCζ interaction with PIP_2_-containing membranes mediated by electrostatic interactions between the anionic PIP_2_ with both the first EF-hand domain and the XY-linker region of PLCζ, which are rich in cationic residues.

## Experimental Procedures

### 

#### 

##### Plasmid Construction

A pCR3-mouse PLCζ-luciferase (PLCζ-luc) construct (21) was subjected to site-directed mutagenesis (QuikChange II, Stratagene) to sequentially generate the three single, one double, and one triple substitutions at Lys-49, Lys-53, and Arg-57, thus producing the PLCζ^K49A^, PLCζ^K53A^, PLCζ^R57A^, PLCζ^K49A,R57A^, and PLCζ^K49A,K53A,R57A^ mutants.

pCR3-PLCζ^K49A,K53A,R57A,K374A,K375A,K377A^-luc construct was generated by a three-step cloning strategy. PLCζEF^K49A,K53A,R57A^ (1–149 amino acids) was amplified from the PLCζ^K49A,K53A,R57A^-luc plasmid by PCR with primers to incorporate a 5-KpnI site and a 3-EcoRI site and then cloned into the pCR3 vector. PLCζΔEF^K374A,K375A,K377A^ (150–647 amino acids) was then amplified from the PLCζ^K374A,K375A,K377A^-luc plasmid (17) with primers to incorporate a 5-EcoRI site and a 3-NotI site in which the stop codon had been removed and cloned into the pCR3-PLCζEF^K49A,K53A,R57A^ plasmid. Finally, luciferase was amplified from pGL2 with primers incorporating NotI sites, and the product was cloned into the NotI site of the pCR3-PLCζ^K49A,K53A,R57A,K374A,K375A,K377A^ plasmid.

All the above PLCζ mutants were amplified from their corresponding pCR3 plasmid with the appropriate primers to incorporate a 5-SalI site and a 3-NotI site, and the products were cloned into the pETMM60 vector to enable bacterial protein expression. The primers used for the amplifications were as follows: 5′-GAACGTCGACATGGAAAGCCAACTTCATGAGCTCGC-3′ (forward) and 5′-GGAAGCGGCCGCTCACTCTCTGAAGTACCAAAC-3′ (reverse). Successful mutagenesis and cloning of the above expression vector constructs were confirmed by dideoxynucleotide sequencing (Applied Biosystems Big-Dye Version 3.1 chemistry and model 3730 automated capillary DNA sequencer by DNA Sequencing & Services^TM^).

##### cRNA Synthesis

Following linearization of wild-type and mutated PLCζ plasmids, cRNA was synthesized using the mMessage Machine T7 kit (Ambion) and then was polyadenylated using the poly(A) tailing kit (Ambion), as per the manufacturer's instructions.

##### Preparation and Handling of Gametes

Female mice were super-ovulated and mature MII eggs were collected from excised oviducts 13.5–14.5 h after injection of human chorionic gonadotrophin and maintained in droplets of M2 media (Sigma) under mineral oil at 37 °C. Experimental recordings of Ca^2+^ release or luciferase expression were carried out with mouse eggs in Hepes-buffered media (H-KSOM), as described previously (22). All compounds were from Sigma unless stated otherwise. All procedures using animals were performed in accordance with the United Kingdom Home Office Animals Procedures Act and were approved by the Cardiff University Animals Ethics Committee.

##### Microinjection and Measurement of Intracellular Ca^2+^ and Luciferase Expression

Mouse eggs were washed in M2 and microinjected with cRNA diluted in injection buffer (120 mm KCl, 20 mm Hepes, pH 7.4). The volume injected was estimated from the diameter of cytoplasmic displacement caused by the bolus injection. All injections were 3–5% of the oocyte volume. Eggs were microinjected with the appropriate cRNA in the injection buffer, mixed with an equal volume of 1 mm Oregon Green 1,2-bis(2-aminophenoxy)ethane-*N*,*N*,*N*′,*N*′-tetraacetic acid-dextran (Life Technologies, Inc.). Eggs were then maintained in H-KSOM containing 100 μm luciferin and imaged on a Nikon TE2000 microscope equipped with a cooled intensified CCD camera (Photek Ltd., UK). The luminescence (luciferase expression) and fluorescence (for Ca^2+^ measurements) from eggs were collected by switching back and forth between the two modes on a 10-s cycle (23, 24). These two signals were then displayed as two separate signals over the same time period for each egg. The fluorescent light used to measure Ca^2+^ is shown in relative units. Luminescence was recorded as photon counts/s and plotted as a running average over 5 min. All live imaging experiments on eggs were made during a 1-month period.

##### Protein Expression and Purification

For NusA-His_6_-fusion protein expression, *Escherichia coli* (BL21-CodonPlus(DE3)-RILP; Stratagene) cells were transformed with the appropriate pETMM60 plasmid and cultured at 37 °C until the *A*_600_ reached 0.6, and protein expression was induced for 18 h at 16 °C with 0.1 mm isopropyl 1-thio-β-d-galactopyranoside (ForMedium). Cells were harvested (6000 × *g* for 10 min), resuspended in PBS containing a protease inhibitor mixture (EDTA-free; Roche Applied Science), and sonicated four times for 15 s on ice. Soluble NusA-His_6_-tagged fusion protein was purified on nickel-nitrilotriacetic acid resin following standard procedures (Qiagen) and eluted with 250 mm imidazole. Eluted proteins were dialyzed overnight (10,000 molecular weight cutoff; Pierce) at 4 °C against 4 liters of PBS and concentrated with centrifugal concentrators (Sartorius; 10,000 molecular weight cutoff).

##### Assay of PLC Activity

PIP_2_ hydrolytic activity of recombinant PLCζ proteins was assayed as described previously (17, 21). The final concentration of PIP_2_ in the reaction mixture was 220 μm, containing 0.05 μCi of [^3^H]PIP_2_. The assay conditions were optimized for linearity, requiring a 1-min incubation of 200 pmol of PLCζ protein sample at 25 °C. In assays to determine dependence on PIP_2_ concentration, 0.05 μCi of [^3^H]PIP_2_ was mixed with cold PIP_2_ to give the appropriate final concentration. In assays examining Ca^2+^ sensitivity, Ca^2+^ buffers were prepared by EGTA/CaCl_2_ admixture, as described previously (17, 21).

##### Protein Lipid Overlay Assay

PIP array membranes (Echelon Biosciences) were blocked for 2 h with binding buffer (TBS-T: 20 mm Tris, 137 mm NaCl, 0.1% Tween 20, pH 7.4) containing 3% bovine serum albumin (lipid-free) and incubated with 25 pmol of each NusA-PLCζ fusion protein for 1 h at room temperature. After washing three times in TBS-T, NusA-PLCζ fusion protein interaction with the inositol phosphate lipids was detected by first incubating the PIP array membranes with penta-His monoclonal antibody (Qiagen, 1:5000 dilution in 5 ml of binding buffer) overnight at 4 °C, followed by three 15-min washes. This was followed by incubation with horseradish peroxidase-conjugated anti-mouse antibody in the same binding buffer for 1 h at room temperature, followed by three 15-min washes with TBS-T. Detection of horseradish peroxidase-coupled secondary antibody was achieved using enhanced chemiluminescence detection (ECL; Amersham Biosciences).

##### Liposome Preparation and Binding Assay

Unilamellar liposomes were prepared as described previously (17, 25) by the extrusion method using a laboratory extruder (LiposoFast-Pneumatic, Avestin Inc., Ottawa, Ontario, Canada) with lipids purchased from Avanti Polar Lipids Inc. (Alabaster, AL). In a typical experiment for preparing a 2-ml dispersion of liposomes, 0.038 mmol (19 × 10^−3^ m) of 1,2-dipalmitoyl-*sn-*glycero-3-phosphocholine (PtdCho), 0.019 mmol (9.5 × 10^−3^ m) of cholesterol (CHOL; molar ratio of PtdCho/CHOL, 2:1), 0.0095 mmol (4.8 × 10^−3^ m) of 1,2-dimyristoyl-*sn*-glycero-3-phosphoethanolamine (PtdEtn; molar ratio of PtdCho/PtdEtn, 4:1), and 5% 1,2-diacyl-*sn*-glycero-3-phospho-l-serine (PS) or 1–5% 1,2-diacyl-*sn*-glycero-3-phosphate sodium salt (PA) or 1% of 1,2-diacyl-*sn*-glycero-3-phospho-(1-d-myo-inositol 4,5-bisphosphate) sodium salt (PIP_2_) were dissolved in a chloroform/methanol solution (2:1 v/v) for the formation of lipid films. The film was hydrated with 2 ml of PBS, and the resultant suspension was extruded through two stacked polycarbonate filters of 100-nm pore size. Twenty five cycles of extrusion were applied at 50 °C. Dynamic light scattering was employed to determine the size of the liposomes, which used a light scattering apparatus (AXIOS-150/EX, Triton Hellas, Thessaloniki, Greece) with a 30-milliwatt laser source and an Avalanche photodiode detector set at a 90° angle. Dynamic light scattering measurements of the extruded lipid preparation showed a narrow monomodal size distribution with average liposome diameter of 100 nm and a polydispersity index of 0.20–0.25. For protein binding studies, liposomes (100 μg) were incubated with 1 μg of recombinant protein for 30 min at room temperature and centrifuged for 5 h at 4 °C. The supernatant and pellet were then analyzed either by SDS-PAGE and Coomassie Blue staining or by the [^3^H]PIP_2_ hydrolysis assay described above.

##### SDS-PAGE and Western Blotting

Recombinant proteins were separated by SDS-PAGE as described previously (25, 26). Separated proteins were transferred onto polyvinylidene difluoride membranes (Immobilon-P; Millipore) using a semi-dry transfer system (Trans-Blot S.D.; Bio-Rad) in transfer buffer (48 mm Tris, 39 mm glycine, 0.0375% SDS, 20% v/v methanol) at 20 V for 1 h. Membranes were incubated overnight at 4 °C in Tris-buffered saline, 0.1% Tween 20 (TBS-T) containing 5% nonfat milk powder, and probed with a penta-His mouse monoclonal antibody (Novagen, 1:100,000 dilution). Detection of horseradish peroxidase-coupled secondary antibody was achieved using enhanced chemiluminescence detection (ECL; Amersham Biosciences).

##### Mathematical Modeling of Oocyte Ca^2+^ Dynamics

Theoretical predictions of the oscillatory Ca^2+^ activity associated with the various PLCζ constructs were provided by a mathematical model of oocyte IP_3_/Ca^2+^ dynamics. The mathematical model, which has previously been presented in detail (25), employs three inter-dependent variables, namely free cytosolic Ca^2+^, Ca^2+^ sequestered in the endoplasmic reticulum, and intracellular concentrations of IP_3_. To account for the specific binding activity of each PLCζ variant, the effective activity of a PLCζ concentration is defined as: *V^e^*_PLC_ = *V*_PLC_·*b*, where *V*_PLC_ is the nominal PLCζ concentration, and *b* is the binding activity estimated experimentally for each construct. Coefficient *b* assumes a value between 0 and 1, whereas *V*_PLC_ can assume values beyond the physiological range when the protein is overexpressed. The mathematical model was coded and numerically integrated on both C++ and a MATLAB platform (MathWorks).

## Results

### 

#### 

##### Effect of EF-hand Mutations on PLCζ-mediated Ca^2+^ Oscillations in Mouse Eggs

**FIGURE 1.**
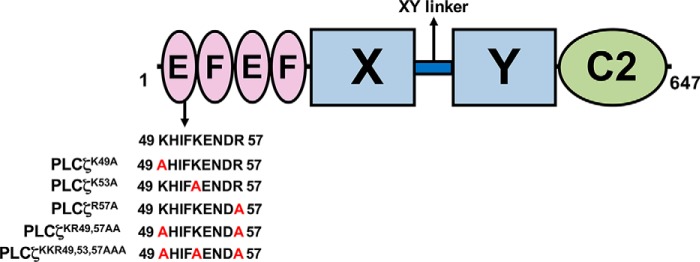
**Generation of PLCζ EF-hand mutants.** Schematic representation of the domain structure of mouse PLCζ identifying the location of the successive Lys or Arg residue substitutions to Ala, between residues 49 and 57 within the first EF-hand domain that are prepared by site-directed mutagenesis for this study.

**FIGURE 2.**
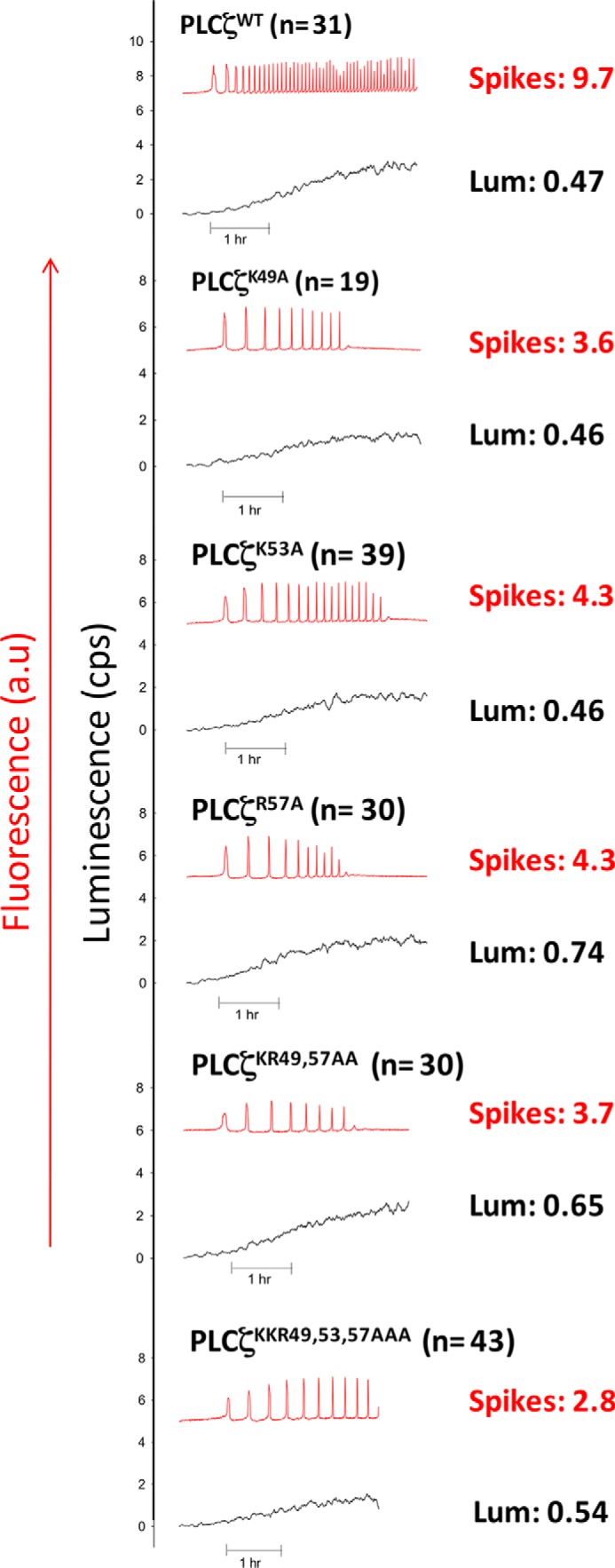
**Expression of wild-type and mutant PLCζ constructs (PLCζ^K49A^, PLCζ^K53A^, PLCζ^R57A^, PLCζ^K53A,K57A^, and PLCζ^K49A,K53A,R57A^) in unfertilized mouse eggs.** Fluorescence and luminescence (*Lum*) recordings reported the Ca^2+^ changes (*red traces*; Ca^2+^) and luciferase expression ((*black traces*; luminescence, in counts/s), respectively, in unfertilized mouse eggs following microinjection of cRNA encoding luciferase-tagged PLCζ constructs. Mean number of Ca^2+^ oscillations in the 1st h of oscillating (*spikes*) and mean luminescence (*cps*) in the 1st h of oscillating (*Lum*) are shown. *a.u.*, arbitrary units.

To investigate the potential importance of a cluster of cationic residues within the first EF-hand unit of the first pair of PLCζ EF-hand domains (Fig. 1), we performed site-directed mutagenesis to produce a panel of cumulative mutations within this positively charged region of the full-length mouse PLCζ. Thus, the residues Lys-49, Lys-53, and Arg-57 were sequentially substituted by the neutral amino acid, alanine, to create three single (PLCζ^K49A^, PLCζ^K53A^, and PLCζ^R57A^) mutants, as well as one double (PLCζ^K53A,K57A^) and one triple (PLCζ^K49A,K53A,R57A^) PLCζ mutant. To test the Ca^2+^ oscillation inducing activity of PLCζ^K49A^, PLCζ^K53A^, PLCζ^R57A^, PLCζ^K49A,R57A^, and PLCζ^K49A,K53A,R57A^ mutants and to verify that these constructs were faithfully expressed as proteins in cRNA-microinjected mouse eggs, we generated C-terminal luciferase-tagged versions of these constructs to enable quantitation of relative protein expression by luminescence detection of the expressed PLCζ-luciferase fusion protein, as described previously (17, 21). Prominent Ca^2+^ oscillations were observed in PLCζ^WT^-luciferase cRNA-injected mouse eggs (9.7 spikes in the 1st h of oscillations) following successful protein expression to a level indicated by a luminescence reading of 0.47 counts/s (Fig. 2 and Table 1), in accord with previous reports (17, 21). Microinjection of cRNA encoding the three single PLCζ mutants (PLCζ^K49A^, PLCζ^K53A^, and PLCζ^R57A^) also triggered Ca^2+^ oscillations (Fig. 2), but these exhibited a lower frequency relative to PLCζ^WT^ (3.6, 4.4, and 4.3 spikes in the 1st h, respectively), although the proteins were expressed at comparable expression levels (Table 1). Similarly, egg microinjection with cRNA encoding either the double PLCζ^K49A,R57A^ or the triple PLCζ^K49A,K53A,R57A^ mutant resulted in a significant reduction in the frequency of Ca^2+^ oscillations compared with PLCζ^WT^, causing 3.7 and 2.8 spikes/1 h, respectively, again when protein was expressed at comparable levels (Fig. 2 and Table 1). These data indicate that the substitution of even one Lys or Arg residue for a neutral Ala within the positively charged cluster of the PLCζ EF-hand domain can significantly alter their Ca^2+^ oscillation inducing activity in mouse eggs by reducing the frequency of Ca^2+^ spikes.

**TABLE 1:** **Properties of PLCζ-luciferase EF-hand mutants expressed in unfertilized mouse eggs** Ca^2+^ oscillation inducing activity (number of Ca^2+^ spikes in the 1st h of oscillations) and luciferase luminescence levels (counts/s of luminescence in 1st h of oscillating) are summarized for mouse eggs microinjected with each of the PLCζ-luciferase mutants as follows: PLCζ^K49A^, PLCζ^K53A^, PLCζ^R57A^, PLCζ^K53A,R57A^, PLCζ^K49A,K53A,R57A^, PLCζ^DMM^, and wild type PLCζ-luciferase (see Figs. 2, 3, and 8*B*). The data are expressed to two significant figures, with means ± S.E. Ratios of total luminescence (counts) per spike in the 1st h of oscillating are also shown expressed to two significant figures with means ± S.E. This is not shown where the number of spikes is zero or where the expression of PLCζ is sufficiently high that the relationship between number of spikes and expression is no longer linear. The results of Mann-Whitney tests for significant differences between the number of spikes for each of the wild-type and mutant PLCζ-luciferase constructs are indicated with *p* values. The results of this same test for a significant difference between the PLCζ mutant ratios (counts/spike) and the wild-type PLCζ-luciferase ratio are also denoted in this way. NA, not applicable.

PLCζ cRNA	No. of eggs	Mean no. of oscillations in 1st h of spiking	Mean expression in 1st h of spiking	No. of spikes significantly different from wild type?	Mean total counts/spike in 1st h of oscillating (counts/spike)	Counts/spike significantly different from wild type?
			*cps*			
PLCζ^WT^	31	9.7 ± 0.63	0.47 ± 0.038	NA	14.61 ± 2.05	NA
PLCζ^K49A^	19	3.6 ± 0.16	0.48 ± 0.024	Yes (*p* = <0.001)	25.92 ± 1.17	Yes (*p* = <0.001)
PLCζ^K53A^	39	4.4 ± 0.13	0.46 ± 0.014	Yes (*p* = <0.001)	20.78 ± 0.85	Yes (*p* = <0.001)
PLCζ^R57A^	30	4.3 ± 0.13	0.74 ± 0.032	Yes (*p* = <0.001)	38.21 ± 2.1	Yes (*p* = <0.001)
PLCζ^K49A,R57A^	30	3.7 ± 0.14	0.65 ± 0.030	Yes (*p* = <0.001)	34.96 ± 1.94	Yes (*p* = <0.001)
PLCζ^K49A,K53A,R57A^	43	2.8 ± 0.074	0.54 ± 0.031	Yes (*p* = <0.001)	37.35 ± 2.24	Yes (*p* = <0.001)
PLCζ^K49A,K53A,R57A^	11	8.6 ± 1.7	7.65 ± 0.92	No (*p* = 0.15)	NA	NA
PLCζ^DMM^	25	0 ± 0	0.53 ± 0.046	Yes (*p* = <0.001)	NA	NA
PLCζ^DMM^	20	0 ± 0	14.43 ± 0.80	Yes (*p* = <0.001)	NA	NA

##### Overexpression of PLCζ^K49A,K53A,R57A^ in Mouse Eggs Rescues Its Defective Ca^2+^ Oscillation-inducing Phenotype

**FIGURE 3.**
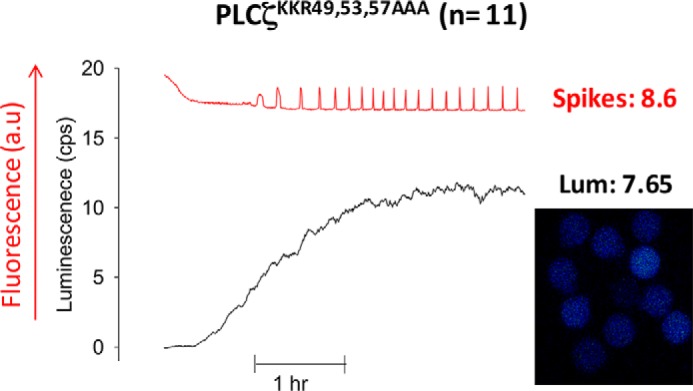
**Overexpression of PLCζ^K49A,K53A,R57A^ in unfertilized mouse eggs.** The *left panel* shows representative fluorescence (*a.u.*, arbitrary units) and luminescence (cps) recordings reporting the Ca^2+^ concentration changes (*red traces*; Ca^2+^) and luciferase expression (*black traces*; *Lum*), respectively, in a mouse egg following microinjection of PLCζ^K49A,K53A,R57A^-luciferase cRNA. The *right panel* shows an integrated image of luciferase luminescence from eggs microinjected with the corresponding PLCζ^K49A,K53A,R57A^-luciferase cRNA for the 1st h of recording. The mean luminescence in the 1st h of oscillating (*Lum*) and mean number of Ca^2+^ spikes in the 1st h of oscillating (*spikes*) are shown. *a.u.*, arbitrary units.

Judging by the number of Ca^2+^ spikes observed within the 1st h of oscillations per unit of recombinant fusion protein expression (cps), PLCζ^WT^ can be seen to be about ∼3.5 times more effective at causing Ca^2+^ oscillations than the PLCζ^K49A,K53A,R57A^ triple mutant. To investigate whether we could rescue the low frequency of Ca^2+^ oscillations induced by PLCζ^K49A,K53A,R57A^, we overexpressed this PLCζ mutant in mouse eggs. As shown in Fig. 3 and Table 1, the overexpression of PLCζ^K49A,K53A,R57A^ (7.65 cps) indeed led to 8.6 spikes in the 1st h of oscillations, comparable with that for PLCζ^WT^, suggesting that loading the egg with large amounts of this PLCζ mutant can rescue its defective Ca^2+^ oscillation-inducing phenotype.

##### Expression and Enzymatic Characterization of PLCζ EF-hand Mutants

**FIGURE 4.**
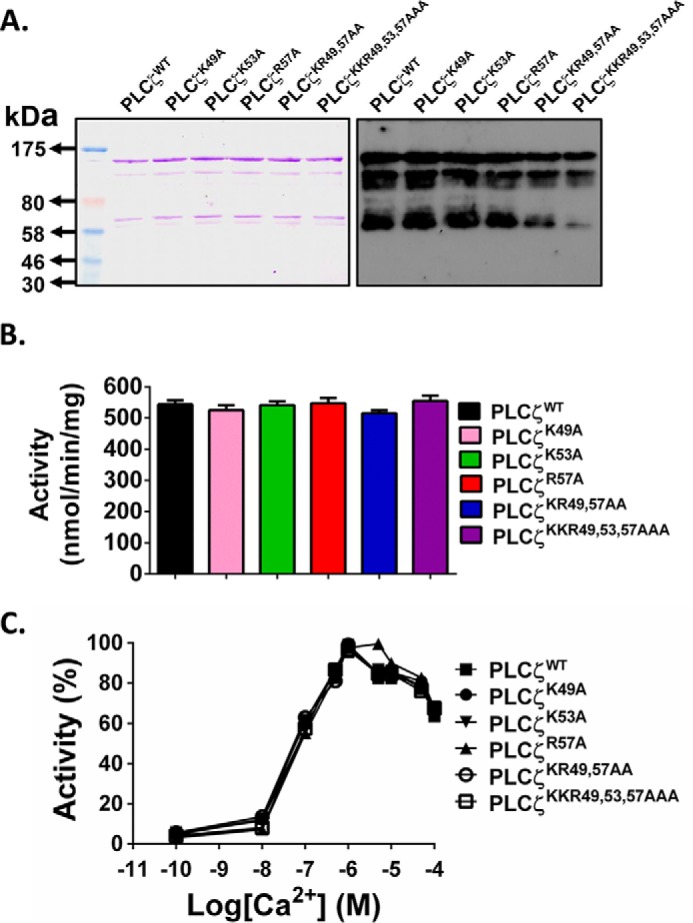
**Expression and enzymatic characterization of recombinant NusA-His_6_-PLCζ EF-hand mutants.** *A,* expression of recombinant NusA-His_6_-PLCζ^WT^ and the various mutant PLCζ proteins. Affinity-purified PLCζ proteins (1 μg) were analyzed by SDS-PAGE followed by either Coomassie Brilliant Blue staining (*left panel*) or immunoblot analysis using the anti-NusA monoclonal antibody at 1:100,000 dilution (*right panel*). *B,* enzyme activity of the various PLCζ mutants. PIP_2_ hydrolysis enzyme activities of the purified NusA-His_6_-PLCζ fusion proteins were determined with the standard [^3^H]PIP_2_ cleavage assay, *n* = 4 ± S.E., using two different preparations of each recombinant protein. An unpaired Student's *t* test showed no significant statistical difference between the enzymatic activities of PLCζ and PLCζ EF-hand mutants. In all cases, *p* > 0.1. *C,* effect of varying [Ca^2+^] on the normalized activity of NusA-His_6_-tagged wild-type and mutant PLCζ fusion proteins. For these assays, *n* = 4 ± S.E., using two different preparations of each recombinant protein.

Each of the PLCζ^K49A^, PLCζ^K53A^, PLCζ^R57A^, PLCζ^K49A,R57A^, and PLCζ^K49A,K53A,R57A^ mutants was subcloned into the pETMM60 vector and purified as NusA-His_6_ fusion proteins by affinity chromatography. We have recently demonstrated that NusA is an effective fusion protein partner for PLCζ, significantly increasing soluble expression of PLCζ protein in *E. coli*, as well as enhancing the enzymatic stability of the purified protein over time (11). Following expression of NusA-PLCζ fusion proteins in *E. coli* and purification by nickel-nitrilotriacetic acid affinity chromatography, samples of each protein were analyzed by SDS-PAGE followed by Coomassie Brilliant Blue staining and immunoblotting using an anti-NusA monoclonal antibody. Fig. 4*A* shows that the major protein band following affinity isolation, with mobility corresponding to the predicted molecular mass of ∼134 kDa for each construct, was present for all fusion proteins analyzed (*left panel*), and these major bands were also recognized in the corresponding anti-NusA immunoblot (*right panel*), confirming the appropriate expression of all PLCζ mutants. Some intermediate molecular mass bands detected by the anti-NusA antibody are the probable result of some degradation occurring through the various protein expression and purification procedures. Similarity of protein expression profile, including degradation products, for the various PLCζ constructs being examined suggests that experimental comparison of relative enzymatic data may be appropriate. Hence, the specific PIP_2_ hydrolytic enzyme activity for PLCζ^WT^ and each recombinant mutant protein was determined by the standard micellar [^3^H]PIP_2_ hydrolysis assay. The histogram of Fig. 4*B* and Table 2 summarize the enzyme specific activity values obtained for each recombinant protein. The enzymatic activities of all recombinant proteins was very similar, suggesting that mutating the basic residues of the first pair of EF-hands to a neutral residue has no effect on the ability of PLCζ to hydrolyze PIP_2_ *in vitro*. Moreover, to investigate the impact of the EF-hand mutations on Ca^2+^ sensitivity of PLCζ enzyme activity, we assessed the ability of these PLCζ recombinant proteins to hydrolyze [^3^H]PIP_2_ at different Ca^2+^ concentrations ranging from 0.1 nm to 0.1 mm. These experiments indicated that there was no significant difference in the Ca^2+^ sensitivity of PIP_2_ hydrolysis for the wild type, and the five EF-hand mutants (Fig. 4*C*) with a very similar EC_50_ value (67–85 nm) displayed by all recombinant PLCζ proteins (Table 2). To compare the enzyme kinetics of wild-type and mutant PLCζs, the Michaelis-Menten constant, *K_m_*, was calculated for each construct (Table 2). The *K_m_* values obtained were similar for human PLCζ^WT^ (84 μm), PLCζ^K49A^ (121 μm), and PLCζ^R57A^ (115 μm), whereas the *K_m_* value for PLCζ^K53A^ (169 μm) and PLCζ^K49A,R57A^ (219 μm) mutants was ∼2- and ∼2.6-fold higher compared with that of PLCζ^WT^. Interestingly, the *K_m_* value for PLCζ^K49A,K53A,R57A^ (432 μm) was ∼5.1-fold higher compared with PLCζ^WT^ (84 μm), suggesting that replacement of these three positively charged residues within the first EF-hand domain affects the *in vitro* affinity of PLCζ for PIP_2_ without affecting the Ca^2+^ sensitivity of this enzyme.

**TABLE 2:** ***In vitro* enzymatic properties of NusA-His_6_-PLCζ EF-hand mutants** Summary of specific enzyme activity and *K_m_* and EC_50_ values of Ca^2+^ dependence for PIP_2_ hydrolysis determined by non-linear regression analysis (GraphPad Prism 5) for the NusA-His_6_ fusion proteins (see Figs. 4 and 9) is shown.

PLC protein	PIP_2_ hydrolysis enzyme activity	Ca^2+^ dependence EC_50_	*K_m_*
	*nmol/min/mg*	*nm*	μ*m*
PLCζ^WT^	544 ± 23	72	84
PLCζ^K49A^	525 ± 28	68	121
PLCζ^K53A^	541 ± 22	75	169
PLCζ^R57A^	547 ± 30	85	115
PLCζ^K49A,R57A^	515 ± 17	67	219
PLCζ^K49A,K53A,R57A^	555 ± 30	79	432
PLCζ^DMM^	434 ± 28	108	4975

##### Binding of PLCζ to PS, PA, and PIP_2_

**FIGURE 5.**
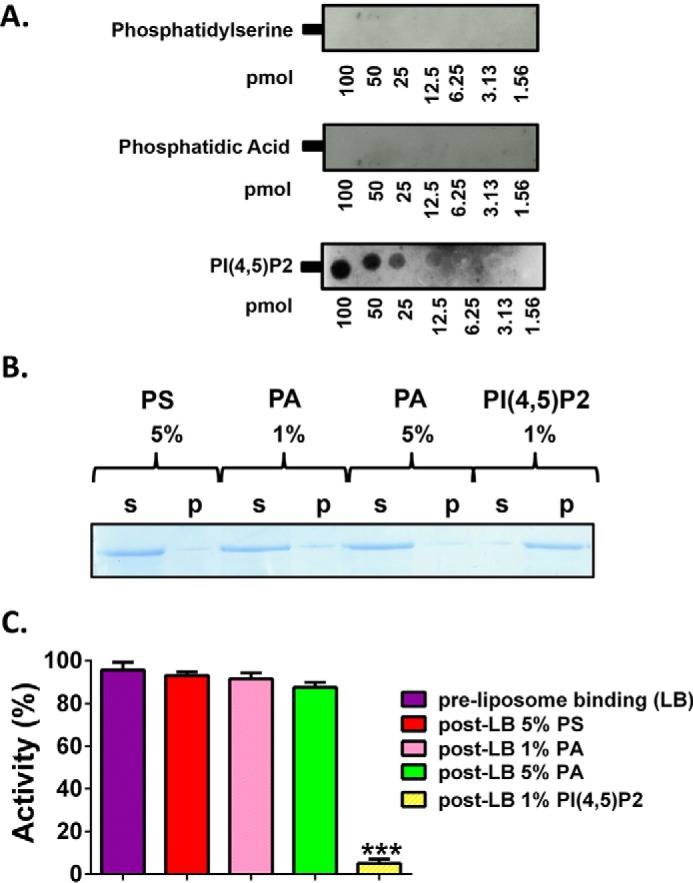
***In vitro* binding of wild-type PLCζ to PS, PA and PIP_2_.** *A,* PLCζ protein-lipid overlay assays. Recombinant protein binding to spotted phospholipids on the PIP arrays was detected using the monoclonal penta-His antibody. *B,* liposome “pulldown” assay of PLCζ. Unilamellar liposomes containing either PS (5%), or PA (1 or 5%), or PIP_2_ (1%) were incubated with PLCζ recombinant protein. Following liposome centrifugation, both the supernatant (*s*) and liposome pellet (*p*) were subjected either to SDS-PAGE and Coomassie Brilliant Blue staining. *C,* supernatants were assayed for their ability to hydrolyze PIP_2_ *in vitro*, using the standard [^3^H]PIP_2_ hydrolysis assay, *n* = 4 ± S.E., using two different preparations of recombinant protein. Significant statistical differences (*asterisks*) were calculated by an unpaired Student's *t* test; ***, *p* < 0.0005 (GraphPad, Prism 5).

To examine the ability of PLCζ to bind the membrane lipids, PS, PA, and PIP_2_, we employed three different approaches. First, we used a protein-lipid overlay assay to assess the binding of PLCζ to membrane-spotted arrays of inositol phospholipids containing PS, PA, or PIP_2_. As shown in Fig. 5*A*, no binding to PS or PA was evident, although PLCζ was able to bind to membrane arrays containing PIP_2_. This result is consistent with our liposome binding assays (Fig. 5*B*). For these binding assays, we made unilamellar liposomes composed of phosphatidylcholine/CHOL/phosphatidylethanolamine (4:2:1) with incorporation of either 5% PS, 1 or 5% PA, and 1% PIP_2_. To diminish any nonspecific protein binding to highly charged lipids, the liposome binding assays were performed in the presence of a near-physiological concentration of MgCl_2_ (0.5 mm). PLCζ displayed robust binding only to liposomes containing 1% PIP_2_, whereas the protein was only detected in the supernatant of liposomes containing 5% PS and 1 or 5% PA (Fig. 5*B*). Finally, we incubated 1 μg of PLCζ recombinant protein with the liposomes composed of the different phospholipids, and after centrifugation, the supernatants were separated and assayed for their ability to hydrolyze PIP_2_ *in vitro*, using the standard [^3^H]PIP_2_ hydrolysis assay. As shown in Fig. 5*C*, only the supernatant obtained after the interaction of recombinant PLCζ protein with the liposomes containing 1% PIP_2_ showed a dramatic ∼94% reduction in its PIP_2_ hydrolytic activity. All these data suggest that the PLCζ binds specifically to PIP_2_, not generically to any anionic phospholipid.

##### Binding of PLCζ EF-hand Mutants to PIP_2_-containing Liposomes

**FIGURE 6.**
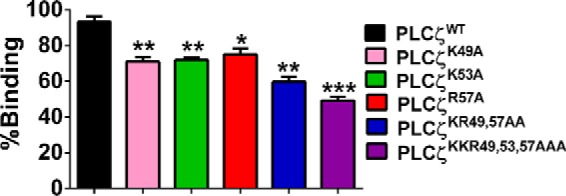
**Binding of PLCζ mutants to PIP_2_-containing liposomes.** Normalized binding of PLCζ^WT^, PLCζ^K49A^, PLCζ^K53A^, PLCζ^R57A^, PLCζ^K53A,K57A^, and PLCζ^K49A,K53A,R57A^ to unilamellar liposomes containing 1% PIP_2_ is shown. Following centrifugation, the supernatants were assayed for their ability to hydrolyze PIP_2_ *in vitro*, using the standard [^3^H]PIP_2_ hydrolysis assay (*n* = 4 ± S.E., using two different preparations of recombinant protein). Based on the percentage of the PIP_2_ hydrolytic activity pre- and post- liposome binding, the relative binding of each PLCζ protein to the PIP_2_-containing liposomes was determined. Significant statistical differences (*asterisks*) were calculated by an unpaired Student's *t* test; *, *p* < 0.05; **, *p* < 0.005; and ***, *p* < 0.0005, (GraphPad, Prism 5).

To investigate the effect of cumulative EF-hand mutations on the PIP_2_-binding properties of wild-type PLCζ, we employed the liposome/activity binding assay as described above (see Fig. 5*C*). Thus, 1 μg of recombinant protein corresponding to PLCζ^WT^ and the five EF-hand mutants were each incubated with liposomes containing 1% PIP_2_. After centrifugation, the supernatants were separated, and the PIP_2_ hydrolytic activity was assayed using the standard [^3^H]PIP_2_ hydrolysis assay. Based on the percentage of the PIP_2_ hydrolytic activity pre- and post-liposome binding, we estimated the relative binding of each PLCζ protein to the PIP_2_-containing liposomes. As shown in Fig. 6, although 94% of PLCζ^WT^ bound to the liposomes, the three single EF-hand mutants (PLCζ^K49A^, PLCζ^K53A^, and PLCζ^R57A^) showed ∼71–75% liposome binding. The effect of the double and the triple mutation was even more notable, as PLCζ^K53A,K57A^ displayed ∼59% and PLCζ^K49A,K53A,R57A^ ∼49% relative liposome binding. These data indicate that sequential neutralization of the basic residues within the EF-hand region substantially reduces the PIP_2_-binding ability of PLCζ.

##### Modeling of Ca^2+^ Oscillations Induced by PLCζ EF-hand Mutants

**FIGURE 7.**
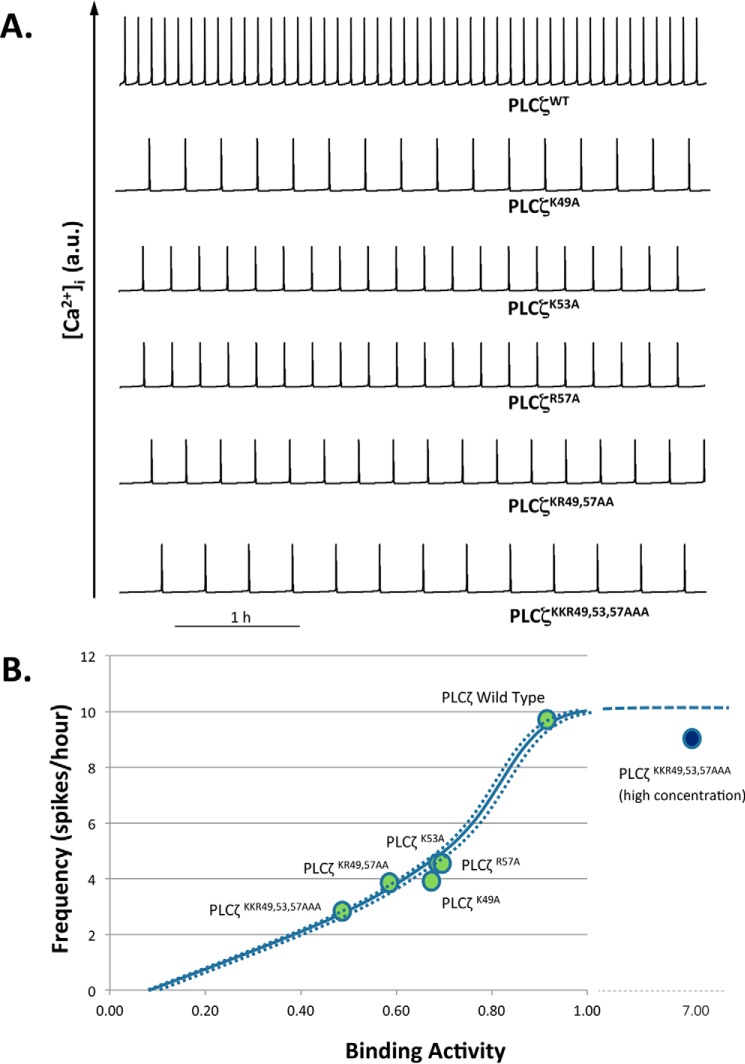
**Simulated time series of Ca^2+^ oscillations within an egg for wild-type and the various PLCζ mutants.** *Top panel,* physiological parameters were taken from experimental measurements summarized in Fig. 6 and Tables 1 and 2. The theoretical relationship between PIP_2_ binding activity of the PLCζ constructs and the Ca^2+^ oscillatory frequency is plotted in the *bottom panel* as a *solid line* for EC_50_ = 75 nm. The *solid curve* is framed by *two dotted lines* corresponding to EC_50_ = 65 and 85 nm (*left* and *right panels*, respectively) to account for the small variability in EC_50_ estimated for the various constructs (indicated by *circles*). The curve is plotted against a normalized range of 0 to 1 to account for the binding activity estimated as a percentile in Fig. 6. Oscillatory activity associated with overexpressed PLCζ^K49A,K53A,R57A^ is indicated by the *solid circle* (*top right*). This point lies below the theoretical binding activity *versus* frequency curve (*dashed line*).

The Ca^2+^ oscillatory activity associated with each of the PLCζ mutants constructed was simulated by using the parameters calculated in Fig. 6 and Tables 1 and 2. The most marked differentiation between constructs is the binding activity of each protein (Fig. 6), which is in agreement with a progressive destabilization of the EF-hand binding regime. By contrast, the Ca^2+^ dependence of IP_3_ production (plotted in Fig. 4 and quantified in Table 2 as Ca^2+^-dependent EC_50_ value) is very similar for each of the PLCζ constructs. Ca^2+^ oscillations simulated with this set of parametric values (Fig. 7, *top panel*) closely match those observed experimentally for each construct (Fig. 2) in terms of frequency. The theoretical relationship between Ca^2+^ oscillatory frequency and binding activity was produced by the mathematical model for EC_50_ = 65, 75, and 85 nm (Fig. 7, *bottom panel,*, *lines left to right*). The experimentally computed operating points of PLCζ wild-type and its various constructs (Fig. 7, *bottom panel, circles*) are located very close to the theoretical curves, confirming that the variability in Ca^2+^ oscillatory frequency can be accounted for almost exclusively by the gradual reduction in binding activity. When PLCζ^K49A,K53A,R57A^ was highly overexpressed, the oscillatory activity was largely restored, as indicated by the operating point of this scenario (Fig. 7, *bottom panel, solid circle* at the *right of the panel*). The fact that the circle lies below the theoretical frequency curve (Fig. 7, *bottom panel, dashed line*) may be due to the sub-optimal binding of the protein to PIP_2_ at non-physiologically elevated concentrations.

##### Ca^2+^ Oscillation Inducing Activity of PLCζ Double Motif Mutant Expressed in Mouse Eggs

**FIGURE 8.**
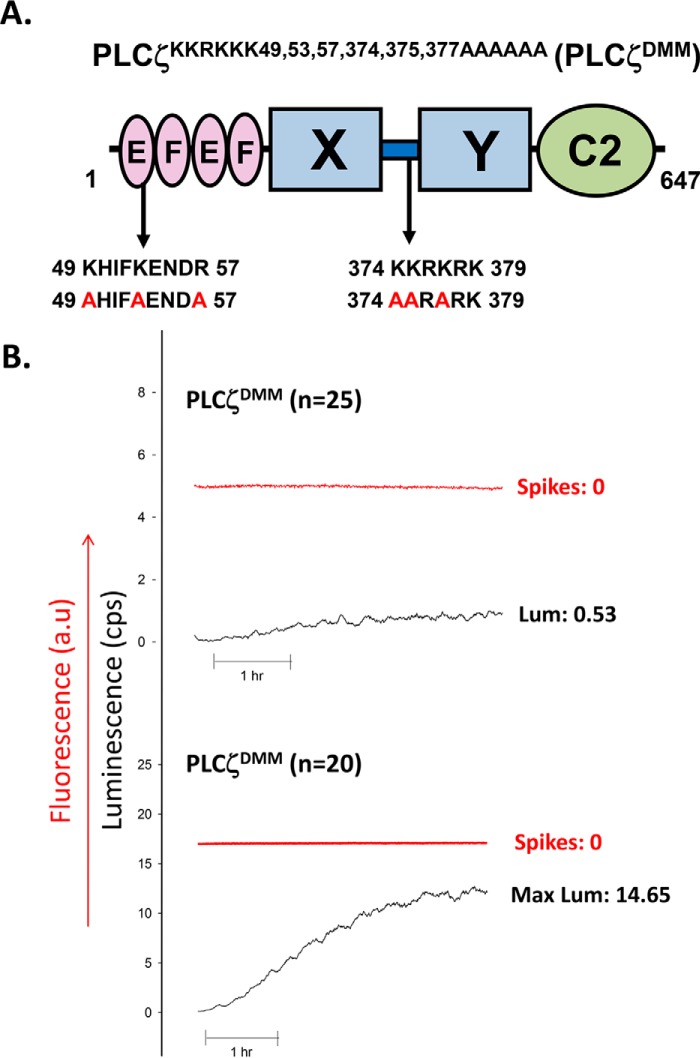
**Generation and expression of PLCζ^K49A,K53A,R57A,K374A,K375A,K377A^ (PLCζ^DMM^) in unfertilized mouse eggs.** *A,* schematic representation of mouse PLCζ domain structure identifying the location of the successive Lys or Arg residue substitutions to Ala, between residues 49 and 57 within the first EF-hand domain, as well as the location of the successive Lys substitutions to Ala between residues 374 and 379 in the XY-linker region. *B,* traces showing the changes in fluorescence (*a.u.*, arbitrary units) and luminescence (cps) denoting alterations in Ca^2+^ concentrations (*red trace*) and luciferase expression (*black trace*), respectively, following the microinjection of high and low concentrations of PLCζ^DMM^-luciferase cRNA into unfertilized mouse eggs. The mean values for the number of Ca^2+^ oscillations (*spikes*) and luminescence (*Lum*) during the 1st h of oscillating are shown.

To investigate whether there is synergy between the cationic residues of the first EF-hand domain and the XY-linker region of PLCζ and whether these residues are necessary and sufficient to anchor this sperm protein to its PIP_2_-containing membranes, we generated a PLCζ mutant, in which charge-neutralization mutations were introduced within these two PLCζ motifs. Thus, the residues Lys-49, Lys-53, and Arg-57 within the first EF-hand domain and the residues Lys-374, Lys-375, and Lys-377 within the XY-linker of PLCζ were substituted by the neutral Ala residue giving rise to a PLCζ double motif mutant (PLCζ^K49A,K53A,R57A,K374A,K375A,K377A^; PLCζ^DMM^) containing six neutralization mutations (Fig. 8*A*). Interestingly, microinjection of cRNA encoding a luciferase-tagged version of PLCζ^DMM^ failed to cause any Ca^2+^ release, even after relatively high levels of protein expression in unfertilized mouse eggs (Fig. 8*B* and Table 1).

**FIGURE 9.**
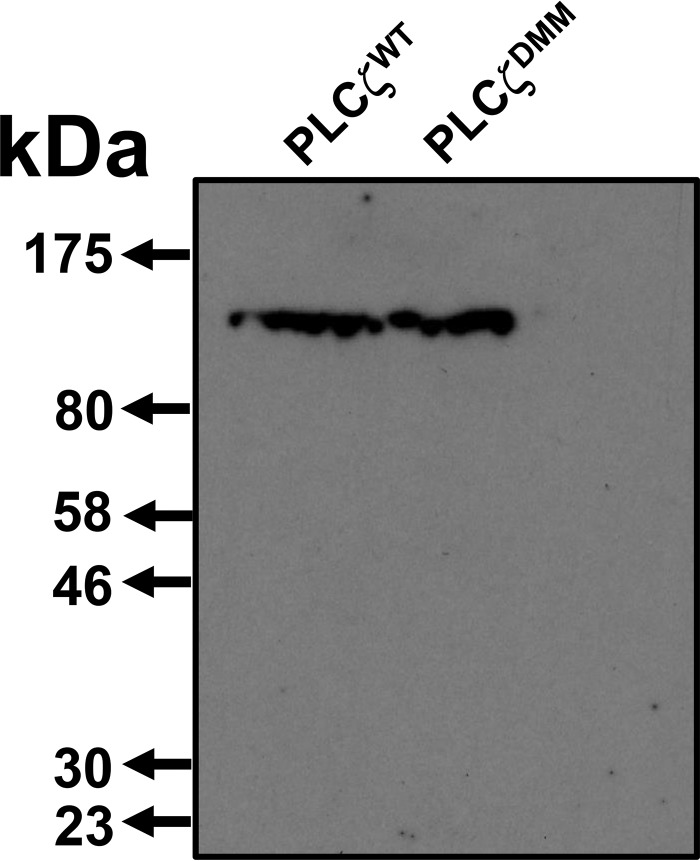
**Confirmation of expression of PLCζ^WT^- and PLCζ^DMM^-LUC fusion proteins in mouse eggs.** Two sets of mouse eggs (50 eggs each) were microinjected with 0.5 μg/μl cRNA corresponding to either PLCζ^WT^ or PLCζ^DMM^. Expression was allowed for ∼3 h, and then the two sets of eggs were analyzed by SDS-PAGE and Western blotting using an anti-firefly luciferase antibody (1:10,000; Pierce).

To investigate whether the luciferase-tagged PLCζ^WT^ and PLCζ^DMM^ fusion constructs were expressed as structurally intact proteins in mouse eggs, we performed immunoblot analysis of two groups of mouse eggs microinjected with 0.5 μg/μl cRNA encoding either PLCζ^WT^-LUC or the PLCζ^DMM^-LUC mutant. Expression was followed for ∼3 h and then the two groups of eggs were analyzed by SDS-PAGE and immunoblot detection using an anti-luciferase antibody. A single protein band was observed with mobility corresponding to the predicted molecular mass (∼129 kDa) for both PLCζ^WT^-LUC and PLCζ^DMM^-LUC fusion proteins (Fig. 9), suggesting that each of the two cRNAs was faithfully expressed as full-length PLC-luciferase proteins and at similar expression levels in the cRNA-injected mouse eggs.

##### Expression, Enzymatic Characterization, and in Vitro Binding of PLCζ Double Motif Mutant to PIP_2_-containing Liposomes

**FIGURE 10.**
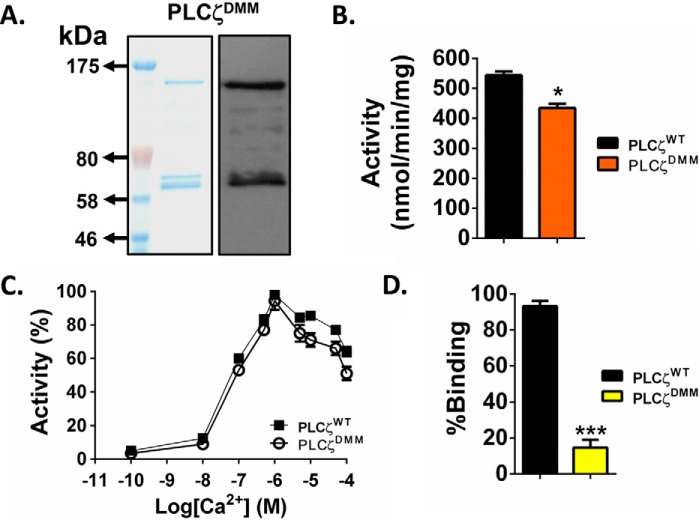
**Expression, enzymatic characterization and *in vitro* binding of NusA-His_6_-PLCζ^DMM^ to PIP_2_-containing liposomes.** *A,* expression of recombinant PLCζ^DMM^ protein. Affinity-purified NusA-His_6_-tagged PLCζ^DMM^ protein (1 μg) was analyzed by SDS-PAGE followed by either Coomassie Brilliant Blue staining (*left panel*) or immunoblot analysis using the anti-NusA monoclonal antibody at 1:100,000 dilution (*right panel*). *B,* enzyme activity of PLCζ^DMM^. PIP_2_ hydrolysis enzyme activities of the purified recombinant proteins were determined with the standard [^3^H]PIP_2_ cleavage assay, *n* = 4 ± S.E., using two different preparations of each recombinant protein. Significant statistical differences (*asterisks*) were calculated by an unpaired Student's *t* test; *, *p* < 0.05 (GraphPad, Prism 5). *C,* effect of varying [Ca^2+^] on the normalized activity of NusA-His_6_-tagged PLCζ^DMM^ fusion protein. For these assays *n* = 4 ± S.E., using two different preparations of each recombinant protein. *D,* normalized binding of PLCζ^DMM^ to unilamellar liposomes containing 1% PIP_2_ (*n* = 4 ± S.E., using two different preparations of recombinant protein). Significant statistical differences (*asterisks*) were calculated by an unpaired Student's *t* test; ***, *p* < 0.0005, (GraphPad, Prism 5).

PLCζ^DMM^ was then subcloned into the pETMM60 vector and bacterially expressed and purified as a NusA-His_6_-tagged fusion protein. Fig. 10*A* shows NusA-His_6_-PLCζ^DMM^ recombinant protein analyzed by SDS-PAGE (*left panel*) and immunoblot detection with the anti-NusA monoclonal antibody (*right panel*). The corresponding protein with the appropriate molecular mass (∼134 kDa) was observed as the top band in both Coomassie Brilliant Blue staining and on the immunoblot (Fig. 10*A*). Some low molecular weight bands were also detected by the anti-NusA antibody, and these are probably the result of protein degradation occurring through the bacterial expression and purification processes. Enzymatic analysis using the [^3^H]PIP_2_ hydrolysis assay showed that PLCζ^DMM^ retained ∼80% of the enzymatic activity of PLCζ^WT^ (434 ± 28 *versus* 544 ± 23 nmol/min/mg) (Fig. 10*B*) and that there was no significant difference in the Ca^2+^ sensitivity of PIP_2_ hydrolysis for PLCζ^WT^ and PLCζ^DMM^, with a very similar EC_50_ value (72 *versus* 108 nm) (Fig. 10*C* and Table 2). However, the *K_m_* value for PLCζ^DMM^ (4975 μm) was ∼59-fold higher compared with PLCζ^WT^ (84 μm). More interestingly, when we performed the liposome/activity binding assay for PLCζ^DMM^, we found that this mutant displayed only ∼15% relative liposome binding compared with PLCζ^WT^ (Fig. 10*D*). These data indicate that neutralization of the positively charged residues within the first EF-hand and the XY-linker region dramatically reduces the binding of PLCζ to PIP_2_, leading to complete loss of its *in vivo* Ca^2+^ oscillation inducing activity.

## Discussion

A significant body of scientific and clinical evidence suggests that the sperm-specific PLCζ protein is the physiological molecule that, following sperm-egg fusion, stimulates cytoplasmic Ca^2+^ oscillations, egg activation, and early embryonic development to effect mammalian fertilization (3, 5, 7, 8, 11, 21, 27). The most compelling observation is that solely introducing PLCζ mimics all of the signaling processes initiated by the sperm, triggering the same pattern of Ca^2+^ release as seen at normal fertilization and leading to the successful development of a blastocyst embryo. Thus, the current model of egg activation at fertilization is that the PLCζ of a fertilizing spermatozoon is introduced into the egg cytoplasm where it catalyzes PIP_2_ hydrolysis, stimulating the IP_3_ signaling pathway, and leading to Ca^2+^ oscillations (5, 13).

The sperm PLCζ is the smallest, with the most elementary domain organization, of all the mammalian PLC isoforms (3). Hence, the intrinsic ability of sperm PLCζ to cause robust Ca^2+^ oscillations in eggs is significant because all the other PI-specific PLCs are unable trigger Ca^2+^ oscillations in eggs at physiological protein expression levels. It therefore appears most plausible that PLCζ employs a novel mechanism to potently induce Ca^2+^ release in eggs and each of its individual domains appears to play an important role in the distinct molecular and biochemical characteristics, as well as in the unique regulatory mechanism of this sperm-derived PLC isozyme (2, 12). PLCζ shares the greatest homology with PLCδ1, but one major structural difference that distinguishes PLCζ from PLCδ1 is the lack of an N-terminal PH domain (2, 13). This is mechanistically interesting because the PH domain of PLCδ1 in particular is known to specifically bind PIP_2_ in the plasma membrane (15, 28). In contrast, we have recently shown that PLCζ does not localize to the plasma membrane-bound PIP_2_, but instead it targets distinct vesicular structures inside the egg cortex (29). Interestingly, the chimeric addition of a PH domain at the N terminus of the PLCζ sequence does not alter the ability of PLCζ to trigger Ca^2+^ oscillations in mouse eggs, and the PH-PLCζ chimera is unable to target PLCζ to the plasma membrane PIP_2_ (25). The precise mechanism employed by PLCζ to enable interaction with the PIP_2_-containing vesicular membranes inside the egg cytosol is not understood.

Although the precise identity of the intracellular PIP_2_-containing vesicles is currently unknown, we have proposed that PLCζ associates with vesicular PIP_2_ via electrostatic interactions mediated by the positively charged XY-linker region, assisting in anchoring PLCζ to membranes, while enhancing local concentrations of the negatively charged PIP_2_ (16, 17). In PLCζ, the XY-linker region is more extended compared with that of PLCδ1, and the proximal part to the Y catalytic domain contains a distinctive cluster of basic amino acid residues not found in the homologous region of any of the other somatic PLC isoforms (3). It is also notable that the XY-linker of somatic PLCs confers potent inhibition of their enzymatic activity (30, 31). In contrast, the XY-linker of PLCζ does not confer enzymatic auto-inhibition but conversely appears to be required for maximal enzymatic activity (19). We have recently shown that deletion of PLCζ XY-linker significantly diminishes its *in vivo* Ca^2+^ oscillation inducing activity but does not completely abolish it (19). This suggests that the XY-linker is essential for the association of PLCζ with PIP_2_-containing vesicular membranes, but it is not the sole region of PLCζ responsible for this association.

Another candidate region that might be involved in the sequestration of PLCζ to membranes containing its substrate PIP_2_ is the C2 domain. The current data indicate that the C2 domain of PLCζ may interact, albeit with low affinity, with membrane phospholipids (17, 32). Indeed, such interactions were observed *in vitro* with phosphatidylinositol 3-phosphate and phosphatidylinositol 5-phosphate. It is possible that the association of the C2 domain with phosphatidylinositol 3-phosphate may play a role in PLCζ localization, or even perhaps regulation of enzymatic activity, as the presence of phosphatidylinositol 3-phosphate reduced PIP_2_ hydrolysis by PLCζ *in vitro* (32).

**FIGURE 11.**
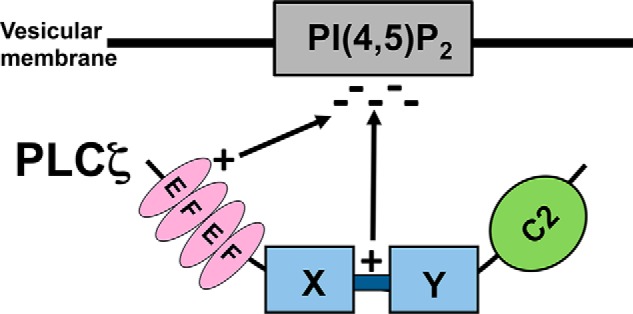
**Schematic illustration of the proposed mechanism that PLCζ utilizes to target intracellular vesicular PIP_2_-containing membranes.** Association of PLCζ with the negatively charged PIP_2_ involves electrostatic interactions with the positively charged first EF-hand domain and the XY-linker region. The catalytic XY domain subsequently proceeds with the enzymatic cleavage of PIP_2_.

A recent study demonstrated that the N-terminal lobe of the EF-hand domain of PLCδ1 binds to anionic phospholipid-containing vesicles, suggesting that the EF-hand domain aids substrate binding in the active site when the protein is membrane-anchored (20). The binding of the PLCδ1 EF-hand domain to anionic phospholipid is mediated by a number of cationic residues within the first EF-hand motif of PLCδ1. Interestingly, the positively charged residues that have been shown to contribute to the binding of PLCδ1 (Arg-182, Lys-183, and Arg-186) by vesicles containing anionic lipids are specifically conserved in PLCζ. We have shown that PLCζ EF-hand domains play an important role in the high Ca^2+^ sensitivity relative to the other PLC isoforms, especially in comparison with PLCδ1 (21). PLCζ appears to be 100-fold more sensitive to Ca^2+^ than PLCδ1, which would enable the enzyme to be active at the resting nanomolar Ca^2+^ levels within the egg cytosol (21). Deletion of one or both pairs of EF-hand domains of PLCζ completely abolishes its Ca^2+^ oscillation inducing activity in mouse eggs (21). Our current data suggest that this might be the result of both altered Ca^2+^ sensitivity and loss of ability to associate with PIP_2_-containing membranes, as these PLCζ EF-hand deletion constructs were unable to trigger Ca^2+^ release even when overexpressed in mouse eggs (21). Our mutagenesis analysis indicates that the substitution of even one Lys or Arg residue to Ala within the positively charged cluster of the PLCζ EF-hand domain diminishes the Ca^2+^ oscillation inducing activity of PLCζ (Fig. 2) without affecting its ability to hydrolyze PIP_2_ *in vitro* or the Ca^2+^ sensitivity of its enzymatic activity (Fig. 4). Interestingly, the *K_m_* value for the triple mutant PLCζ^K49A,K53A,R57A^ (432 μm) was ∼5.1-fold higher compared with PLCζ^WT^ (84 μm), suggesting that replacement of these three positively charged residues within the first EF-hand domain has an effect on the *in vitro* binding ability of PLCζ to PIP_2_ (Table 2). Moreover, we used a variety of approaches and demonstrated that PLCζ binds only to PIP_2_-containing liposomes, and sequential neutralization of these basic residues within the first EF-hand region of PLCζ can significantly diminish the PIP_2_-binding ability of PLCζ (Figs. 5 and 6). As shown in our proposed mechanism in Fig. 11, which is supported by our studies on the PLCζ^K49A,K53A,R57A,K374A,K375A,K377A^ mutant (PLCζ^DMM^), it is plausible that PLCζ is attracted to the anionic PIP_2_-containing component of the intracellular vesicular membranes through electrostatic interactions with both the first EF-hand domain and the XY-linker regions, which are rich in basic residues.

Our study provides an important advance in understanding the complex regulatory mechanism of PLCζ and suggests that the N-terminal lobe of the EF-hand domain of PLCζ has an essential role in the interaction of this enzyme with its target membrane, which together with the XY-linker may combine to provide a tether that facilitates proper PIP_2_ substrate access and binding in the PLCζ active site.

## Author Contributions

M. N., K. S., and F. A. L. designed the study; J. R. S. conducted the oocyte experiments; D. P. generated the simulation data; M. N., L. B., B. L. C., P. S., A. S., and M. C. performed the molecular cloning, protein expression, purification, and characterization experiments; Z. S. prepared the liposomes, and all authors contributed to manuscript preparation. M. N. compiled the figures and together with F. A. L. prepared the final draft.

## Footnotes

3 The abbreviations used are:

PLCζ: phospholipase C-ζ
PIP_2_: phosphatidylinositol 4,5-bisphosphate
IP_3_: inositol 1,4,5-trisphosphate
PH: pleckstrin homology
PtdCho: 1,2-dipalmitoyl-*sn-*glycero-3-phosphocholine
CHOL: cholesterol
PtdEtn: 1,2-dimyristoyl-*sn*-glycero-3-phosphoethanolamine
PS: phosphatidylserine
PA: phosphatidic acid.
